# Comparison of lateral tail vein and retro-orbital venous sinus as routes of inoculation to study Group B streptococcal systemic infection

**DOI:** 10.1128/spectrum.02104-24

**Published:** 2024-11-29

**Authors:** Jéssica da Conceição Mendonça, Stephen Lumsdaine, Lindsey R. Burcham

**Affiliations:** 1 Department of Microbiology, University of Tennessee, Knoxville, Tennessee, USA; 2 Department of Obstetrics and Gynecology, University of Tennessee Health Sciences Center College of Medicine, Knoxville, Tennessee, USA; The Ohio State University College of Dentistry, Columbus, Ohio, USA

**Keywords:** animal models, systemic infection, *Streptococcus*, GBS

## Abstract

Murine models are commonly used to understand pathogen and host determinants of systemic infection. While these models have proven beneficial for uncovering bacterial mechanisms required for progression to invasive disease, it can be challenging to draw comparisons across studies as several different routes of infection are standardly used for these experiments. In this study, one of the leading bacterial meningeal pathogens, *Streptococcus agalactiae*, or Group B *Streptococcus* (GBS), was used to compare experimental outcomes of two commonly used routes of hematogenous infection, lateral tail vein injection and retro-orbital venous sinus injection. Here we demonstrate that both routes of infection result in systemic disease and the onset of clinical symptoms of infection. We show that retro-orbital venous sinus injection results in an initial increase in bacterial dissemination to the spleen and brain tissue of GBS-infected mice, while an increased bacterial burden was only detected in brain tissues at a later time point. Despite differences in initial dissemination and brain bacterial burden, we found that the route of infection did not significantly impact bacterial burden in the blood, kidney, spleen, heart, and lung tissues at experimental endpoints; and similarly did not impact animal health scores during infection; cytokine and proinflammatory protein abundance in the brain tissue; or overall animal survival. In summary, these findings suggest that both tail vein injection and retro-orbital venous sinus injection are viable models to study Group B streptococcal systemic infection and result in largely similar disease outcomes within our tested parameters.

**IMPORTANCE:**

*Streptococcus agalactiae* or Group B *Streptococcus* (GBS) is the leading cause of invasive disease in neonates and immunocompromised adults and is implicated in severe cases of sepsis, pneumonia, and meningitis. Established murine models of hematogenous systemic infection allow for better understanding and investigation of bacterial dispersion, pathogenesis, meningeal inflammation, and interaction with the host. Here we compared two routes of infection, intravenous lateral tail vein and retro-orbital venous sinus, demonstrating that similar experimental outcomes can be observed, regardless of the route of infection for GBS, specifically. These findings help to reinforce the utility of different systemic infection models and provide insight into comparisons across different established models and how these models can be applied in microbial research.

## INTRODUCTION

Animal models are an integral component of biomedical research and can provide important information to aid in better understanding the biological processes involved in human and animal health (1). Developing best practices and choosing appropriate animal models ensure that data are reliable and reproducible and that animal standards of care are maintained. Rodent models are frequently used for studying human infections and are primarily chosen due to their genetic, anatomic, and physiological similarities to humans; ease of genetic manipulation; accessibility; and relative cost effectiveness (2, 3).

Models investigating systemic microbial infection are often inoculated *via* hematogenous routes (2–7). Established hematogenous systemic infection models reproduce transient bacteremia and allow microbes to disseminate through the circulatory system and proximal organs, and provide a better understanding of bacterial dispersion, pathogenesis, inflammation, immune clearance, and interaction with the host. Two models for studying intravenous routes of infection are lateral tail vein injection and retro-orbital injection (8, 9). Lateral tail vein injections (IV) are the most common route of injection but can prove time-consuming, technically challenging with a high risk of failure, and laborious to perform (10). Retro-orbital venous sinus injections (RO) are growing in popularity as a route of inoculation for studying drug delivery and infectious diseases and can be used as a model that may provide a more humane technique and consistent target (10–13). Previous studies have investigated differences between these hematogenous routes in drug delivery and antibody administration (11, 14–16); however, comparisons of intravascular delivery of pathogens to assess efficient host dissemination and disease onset, to our knowledge, have not been performed.

Here we compared bacterial dissemination, disease progression and survival, and the production of host proinflammatory markers in animals challenged *via* two routes of infection, using the Gram-positive opportunistic pathogen, *Streptococcus agalactiae*, or Group B *Streptococcus* (GBS). Though often present as a commensal species found in the gastrointestinal, upper respiratory, or vaginal microflora of healthy individuals, GBS is also a primary causative agent of bacteremia, sepsis, and meningitis in neonates, and is associated with adult infections such as skin and soft tissue infection, particularly in the context of diabetic foot ulcers and adult urinary tract infections (17–19). Our findings show that GBS may disseminate more rapidly in animals challenged RO versus IV at early timepoints but the onset of symptoms of invasive disease and trends in survival occur at similar rates between both challenge groups. These findings help to reinforce the utility of different systemic infection models and provide insight into comparisons across established models and how these models can be applied in microbial research.

## RESULTS

**Fig 1:**
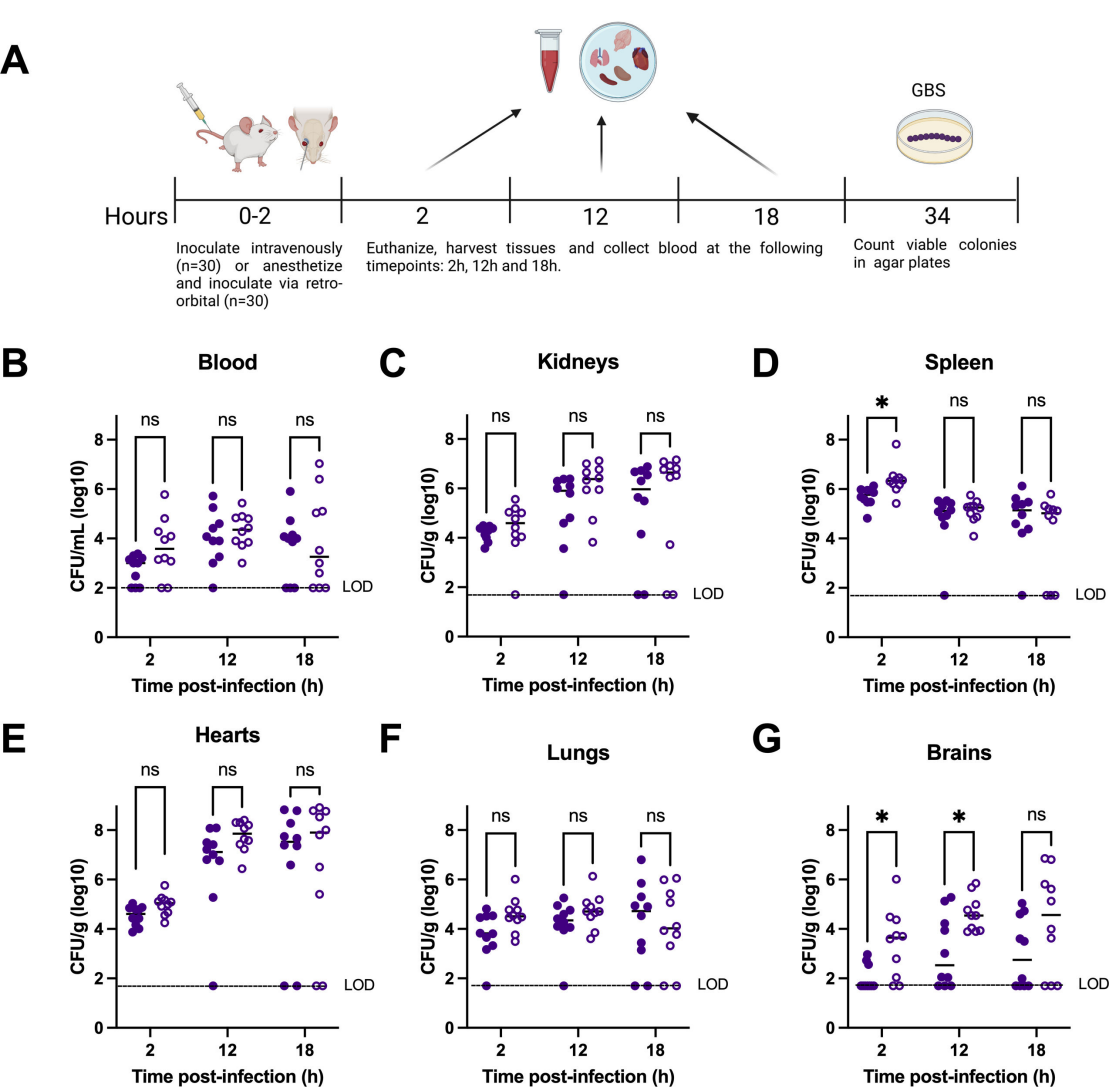
Streptococcal dissemination from IV v. RO route of inoculation. (**A**) Female ICR mice were challenged via IV (*n* = 30, closed circles) or RO (*n* = 30, open circles) with 1 × 10^7^ CFU/100 µL of GBS CJBIII. Animals were sacrificed at 2, 12, and 18 hours post-injection and GBS was recovered from (**B**) blood, (**C**) kidney, (**D**) spleen, (**E**) heart, (**F**) lung, and (**G**) brain. Statistical analyses were determined using the Mann-Whitney U-test. Statistical significance was accepted when *P* < α, with α = 0.05, *P < 0.05 and ns = not significant. The data presented are combined from two independent experiments. LOD: limit of detection.

To determine how route of infection impacts initial GBS dissemination, we assessed systemic bacterial dissemination over time in mice administered GBS *via* lateral tail vein injection (IV) or retro-orbital sinus injection (RO) (Fig. 1A). At 2 hours post-injection, we observed a significant increase in bacterial burden in the spleen and brain tissues of mice infected *via* RO injection compared to those challenged *via* IV injection (Fig. 1D and G). At a later timepoint of 12 hours post-injection, we recovered increased GBS from the brain tissues of mice-infected RO compared to those infected IV (Fig. 1G), while at 18 hours post-injection, no significant differences in GBS tissue burden were observed between mice inoculated IV versus RO (Fig. 1B through G).

**Fig 2:**
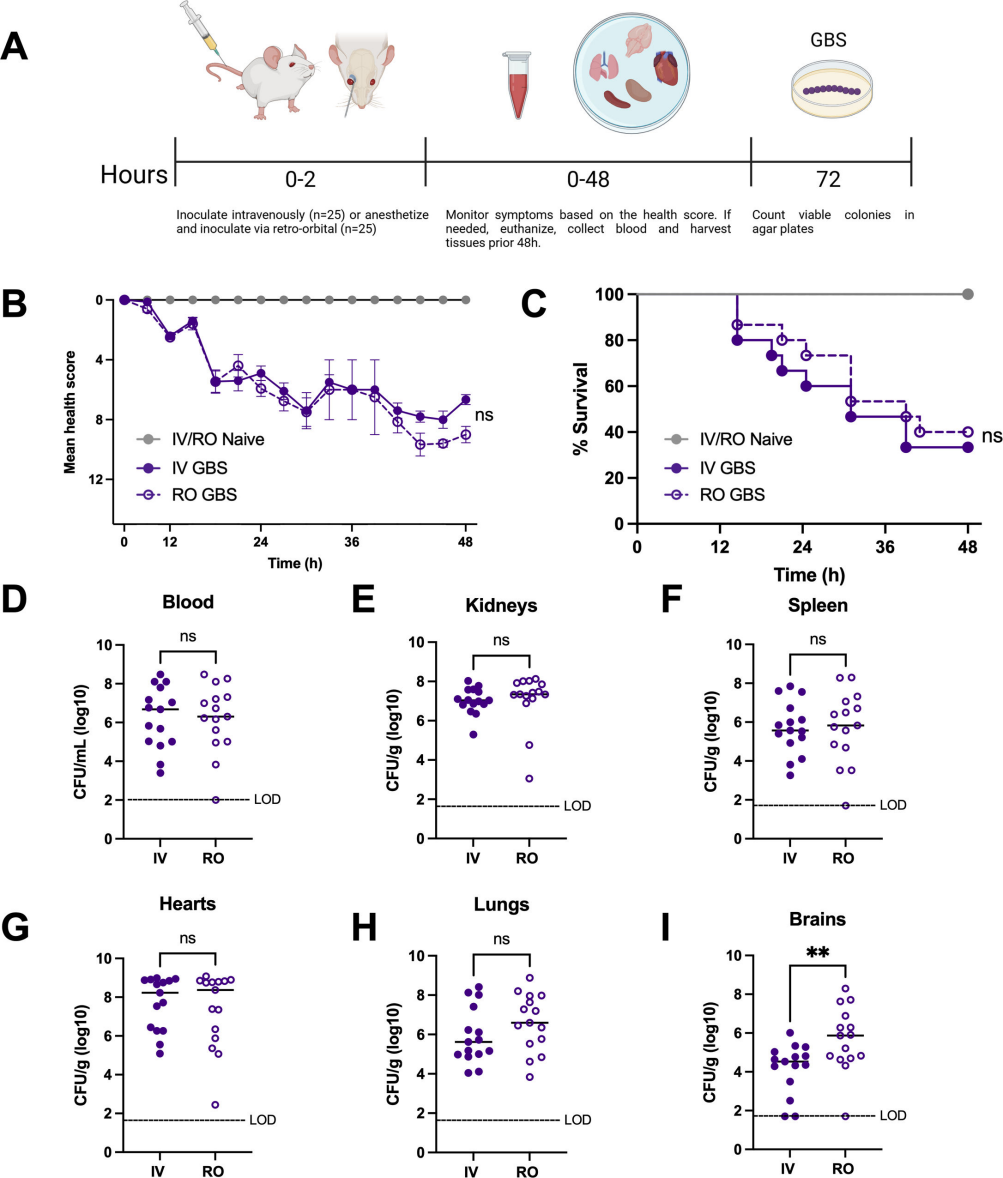
Comparison of IV v. RO inoculation in survival during systemic infection. (**A**) Female ICR mice were challenged *via* IV (*n* = 15, closed circles) or RO (*n* = 15, open circles) with 1 × 10^7^ CFU/100 µL of GBS CJBIII. Naïve controls were included for each route of infection at *n* = 10/group. Kaplan-Meier plots showing (**B**) health scores and (**C**) corresponding survival of mice challenged with GBS via IV (solid lines) or RO (dashed lines) injection. Recovered GBS were quantified from (**D**) blood, (**E**) kidney, (**F**) spleen, (**G**) heart, (**H**) lung, and (**I**) brain. The data presented are combined from two independent experiments. Statistical analyses were determined using the Mantel-Cox Log-rank test (**B and C**) and Mann-Whitney U-test (**D–I**). Statistical significance was accepted when *P* < α, with α = 0.05, *P < 0.05, **P < 0.01, and ns = not significant. LOD: limit of detection.

To investigate how the route of infection may contribute to disease progression, we challenged mice with GBS by IV or RO injection and monitored for the onset of clinical symptoms. Mice were humanely euthanized when deemed moribund or at the experimental endpoint of 48 hours post-injection. At the time of sacrifice, blood and tissues were harvested, homogenized, and plated to assess bacterial burden (Fig. 2A). We observed onset of significant clinical symptoms around 6 hours post-injection in both treatment groups, with a gradual increase in clinical score detected throughout the course of infection (Fig. 2B). Within the first 24 hours post-injection, we observed a nearly 15% initial increase in lethality observed in mice challenged *via* IV compared to mice challenged *via* the RO route of infection; however, this phenotype resolved over the second half of the study (Fig. 2C). Despite the initial increase in bacterial dissemination observed in mice challenged *via* the RO route of infection (Fig. 1), IV and RO-challenged mice developed symptoms of systemic infection and succumbed to infection at an overall similar rate, regardless of the route of infection (Fig. 2B and C). In addition, no significant differences were detected in GBS CFU recovered from mice challenged *via* IV or RO in the blood or kidney, spleen, heart, and lung homogenates harvested at the time of sacrifice (Fig. 2D through H). Interestingly, despite no differences in onset of clinical symptoms or overall survival, we observed a 1.4-fold increase in mean GBS burden in the brain tissues of RO-infected mice compared to those infected IV (Fig. 2I).

**Fig 3:**
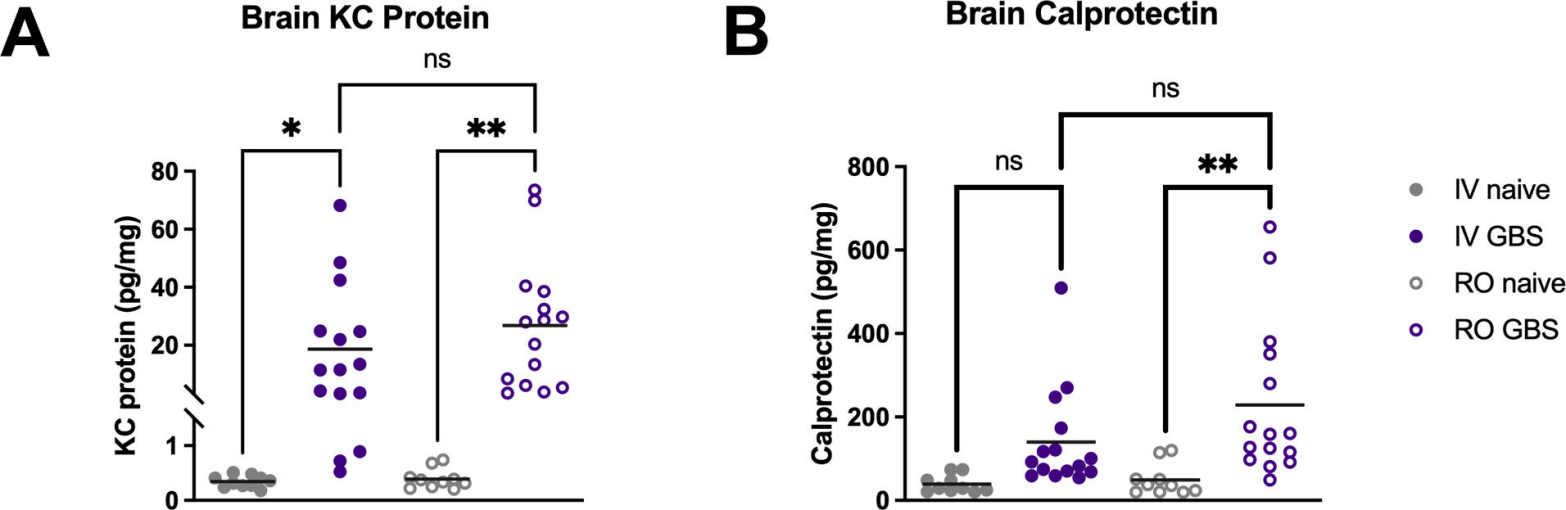
Quantification of inflammatory markers in brain tissue during systemic infection. Protein abundance of KC protein (A) and calprotectin (B) was quantified from brain tissue homogenates by enzyme-linked immunosorbent assay. The data presented are combined from two independent experiments. Statistical analyses were determined using one-way ANOVA with Tukey’s multiple comparisons test (A** and **B). Statistical significance was accepted when *P* < α, with α = 0.05, * *P* < 0.05, **P < 0.01, and ns = not significant.

To assess the impact of the route of infection on inflammatory signaling, we measured the production of the neutrophil recruitment chemokine, KC protein, and the proinflammatory neutrophil marker S100A8/A9, also known as calprotectin, in brain tissue homogenates. We detected significant increases in KC protein in GBS-infected animals compared to the naïve controls, with no statistically significant difference observed between KC levels of mice injected IV versus RO (Fig. 3A). We observed a significant increase in calprotectin expression in GBS-infected mice injected *via* the RO route compared to naïve controls, and though we observed a 3.6-fold mean increase in GBS-infected mice injected IV, this did not reach statistical significance when compared to the IV-injected naïve controls (*P* = 0.2064, Fig. 3B). Brain tissues of mice infected with GBS had similar concentrations of calprotectin regardless of route of infection. Concentrations of proinflammatory markers in the brain tissues were shown to correlate with bacterial burden in mice injected *via* the RO route (**A**) KC protein Spearman r = 0.5464,*P = 0.0377 and (**B**) calprotectin Spearman r = 0.7071, **P = 0.0042), while no correlation was detected in brain tissues of animals challenged via the IV route (**A**) KC protein Spearman r = 0.4415, *ns P* = 0.1007 and (**B)** calprotectin Spearman r = 0.3253, *ns P* = 0.2351) (Fig. S1).

## DISCUSSION

Animal models are an important aspect of biomedical research as they provide meaningful information about biological processes of human health and disease. Hematogenous systemic models of infection are commonly used to assess microbial virulence, animal survival, and host response to infection, and to test potential therapeutic targets or treatment protocols (2). Lateral tail vein injection and retro-orbital (RO) injection serve as two common routes of infection for studying systemic infection. Tail vein injections, though commonly used, can be difficult, require extensive practice, and if performed incorrectly, can lead to a high failure rate and can result in animal distress, which can ultimately impact research outcomes (10). The RO sinus is rich in capillary microcirculation and injections *via* the RO route can lead to successful absorption (15) and can serve as an advantageous and reproducible model for systemic microbial infection in clinically relevant pathogens including *Streptococcus pneumoniae* and *Staphylococcus aureus* (12, 13). In addition, RO injection may serve as a particularly useful murine model for studying pediatric meningeal pathogens such as GBS and *S. pneumoniae*, as it can be adapted for use in neonatal models using pups as young as 1–2 days old (10, 20, 21), where tail vein injection would not be possible. While some studies suggest that RO injections may improve the delivery of drugs and other substances (10), this practice is sometimes discouraged, primarily due to concerns regarding personnel training. To overcome this issue, detailed protocols have been published, including visual resources and description of the behavioral effects of mice during and after RO injections (10, 12, 16), providing auxiliary support to perform this technique.

A previous study assessed lateral tail vein and RO injections as routes of systemic delivery of a therapeutic drug to deplete murine macrophages (11). They observed similar tissue morphology and depletion efficacy regardless of the route of delivery and, based on qualitative observations prior and post-administration, reported substantial indications of behavioral changes between groups. Animals challenged *via* the lateral tail vein showed increased aggression and grooming following injection, while this was not observed in animals that received the drug *via* RO injection. Additional studies comparing lateral tail vein versus RO injection have reported both routes of delivery as sufficient for delivery of radiotracers for positron emission tomography (PET) imaging (15), administration of contrast agent for dynamic contrast-enhanced magnetic resonance imaging (MRI) (22) and injection of radiolabeled cells and antibodies (14).

While numerous studies involving hematogenous animal infection models have provided data on microbial factors involved in disease and potential therapeutic targets to combat systemic and meningeal pathogens, individual studies are often performed across different models, and it is unclear how comparison across models may impact data interpretation. We therefore used GBS, a primary meningeal pathogen and a leading cause of neonatal morbidity and mortality worldwide, to show that inoculation *via* lateral tail vein injection versus RO injection resulted in similar dissemination, development of clinical symptoms, animal survival and bacterial burden, and host signaling. We recovered significantly more GBS from brain tissues at early timepoints and during the final stages of disease in mice challenged *via* RO sinus compared to tail vein (Fig. 1G and 2I). This is likely due to the dense blood flow in the RO sinus, including the supraorbital vein which may contribute to more direct and efficient access to the brain (10), while the lateral tail vein route requires flow through several systemic organs that could serve as an additional physical bottleneck for brain access (23). We also observed significant associations between CFU burden and proinflammatory cytokine production in brain tissues of mice challenged *via* RO but not tail vein. We speculate the abundance of proinflammatory cytokines but lower GBS burden in the brains of mice injected via tail vein may be indicative of a sepsis disease pathology and could have contributed to the initial increase in lethality that we observed near the 24-hour timepoint in the group injected *via* tail vein (Fig. 2C), or potentially due to the sustained exposure to GBS in the RO-inoculated group, as evidence by the robust initial brain dissemination (Fig. 1G).

Though we observed initial differences in bacterial dissemination, both models result in similar disease progression and survival outcomes, and collectively, our findings suggest that lateral tail vein injection and RO venous sinus injection both serve as viable models to study GBS systemic infection. Future studies including comparison of sex as a biological variable, additional experimental models, other microbial pathogens, and evaluation of additional parameters such as tissue histopathology and immune profiling could provide more insight for researchers drawing conclusions across models and into best practice for performing murine models of infection.

## MATERIALS AND METHODS

### Study approval

All experiments were performed under the approval of the Institutional Biosafety Committee (#22-580-2). Animal experiments were conducted under the approval of the Institutional Animal Care and Use Committee (#2937-1222) at the University of Tennessee and performed using accepted veterinary standards.

### Bacterial strains and growth conditions

GBS serotype V neonatal clinical isolate CJB111 (24) was grown at 37°C overnight in Todd-Hewitt Broth (THB, BD Diagnostics) and sub-cultured into THB the morning of experiments. Prior to the start of experiments, GBS CJB111 was grown to the mid-log phase (between 0.4 and 0.5 OD_600 nm_), centrifuged, and the bacterial pellet resuspended in 1 mL of phosphate-buffered saline (PBS). Concentrated culture was used to normalize bacterial inoculum to an OD_600 nm_ 0.4 (~1 ×10^8^ CFU/mL) in PBS.

### Murine models of systemic infection

We utilized two distinct models of hematogenous infection as described previously (7, 10).

For studies investigating the dissemination of GBS, a total of 60 female 8-week-old ICR mice (Envigo/Inotiv) were randomly divided into six groups of ten animals per group – IV 2, 12, and 18 hours and RO 2, 12, and 18 hours. For survival studies, a total of 50 female 8-week-old ICR mice were randomly divided into four groups—IV naïve (*n* = 10), IV-GBS infected (*n* = 15), RO naïve (*n* = 10), and RO-GBS infected (*n* = 15). Mice receiving IV injections were placed in a brass restrainer (Braintree Scientific Inc) and a warm compress was used to promote vascular dilation in the tail. Alcohol pads were used to sterilize the tail and mice were inoculated intravascularly with 1 × 10^7^/CFU GBS CJB111 in a 100 µL volume.

Mice receiving RO injections were anesthetized using isoflurane USP (Vet One) through an isoflurane Tec 3 vaporizer (Vetamac Anesthesia). Once mice were anesthetized and a drop of proparacaine USP (Sandoz) was placed on the eye. Injections were performed with 1 × 10^7^/CFU GBS CJB111 in a 100 µL volume as previously described (25), with the needle inserted on the right medial cantus of the eye, using 28 G x ½″ U100 Insulin Micro-Fine IV injection needles (BD). After injection, a disposable swab was used to treat the eye with a lubricant eye ointment (Allergan) and mice were monitored as they recovered from anesthesia. All mice were carefully inspected and monitored until the 48-hour experimental timepoint. Disease progression was evaluated based on a symptom score checklist accounting for changes in grooming, movement/gait, righting reflex, feeding/drinking, breathing effort, and onset of neurological symptoms. For dissemination studies, mice were sacrificed at 2, 12, and 18 hours post-injection and blood, kidney, spleen, heart, lung, and brain tissues were harvested. Tissues were homogenized and all samples were serially diluted and plated on Todd Hewitt Agar to enumerate bacterial burden.

### Enzyme-linked immunosorbent assays

KC protein and S100A8/A9 from mouse brain homogenates were detected by enzyme-linked immunosorbent assay (R&D Systems) according to the manufacturer’s instructions. Protein concentrations displayed were normalized to tissue weight and dilutions prior to enzyme-linked immunosorbent assay (ELISA). Tissue homogenate dilutions ranged from 1:10-1:40 for calprotectin and 1:3-1:50 for KC protein.

### Statistical analysis

Data analysis was performed using GraphPad Prism version 10.4 (GraphPad Software, San Diego, CA, USA). Statistical significance and specific tests are indicated in the figure legends.

## SUPPLEMENTAL MATERIAL

Figure S1 spectrum.02104-24-s0001.pdf Correlation between GBS bacterial burden and (A) KC protein and (B) calprotectin abundance in brain tissues of IV v.

ASM does not own the copyrights to Supplemental Material that may be linked to, or accessed through, an article. The authors have granted ASM a non-exclusive, world-wide license to publish the Supplemental Material files. Please contact the corresponding author directly for reuse.
