# Supplementary material for: Comparison of lateral tail vein and retro-orbital venous sinus as routes of inoculation to study Group B streptococcal systemic infection
Source: Microbiol Spectr. 2024 Nov 29;13(1):e02104-24. doi: 10.1128/spectrum.02104-24 (PMC11705913; doi:10.1128/spectrum.02104-24)
Supplement: Figure S1 — Correlation between GBS bacterial burden and (A) KC protein and (B) calprotectin abundance in brain tissues of IV v. [file spectrum.02104-24-s0001.pdf]

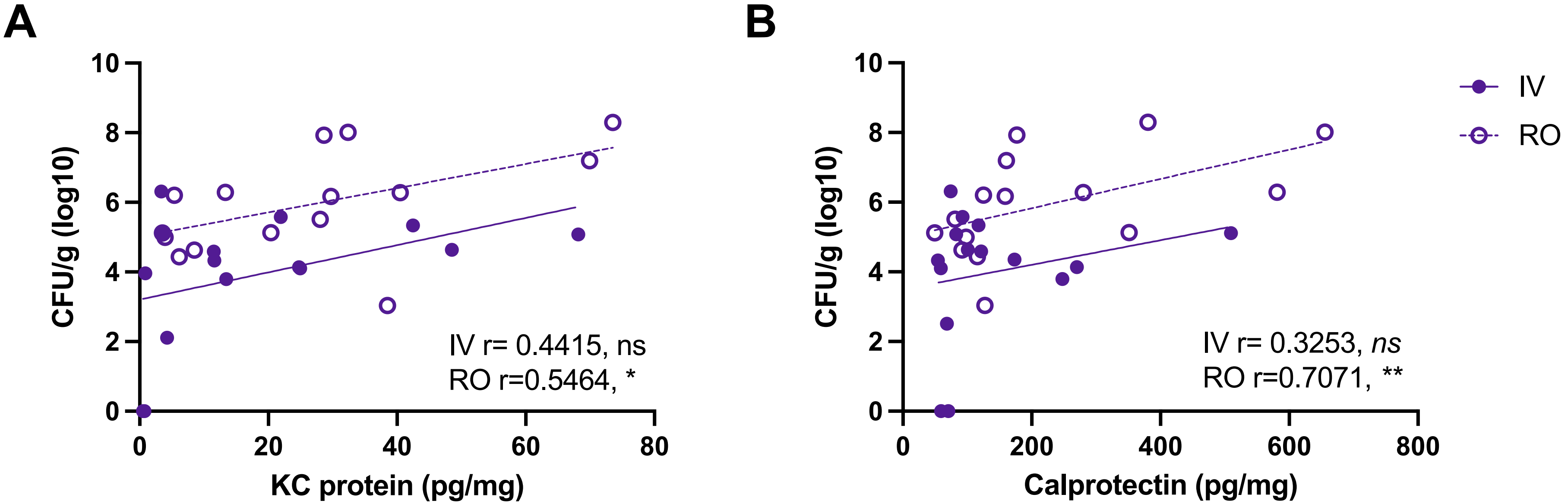

Supplemental Figure 1. Correlation between GBS bacterial burden and **(A)** KC protein and **(B)** calprotectin abundance in brain tissues of IV v. RO-injected animals. Statistical analyses were determined using Spearman correlation calculated for **(A)** KC protein (IV r = 0.4415, *ns* $p = 0.1007$; RO r = 0.5464, * $p = 0.0377$) and **(B)** calprotectin (IV r = 0.3253, *ns* $p = 0.2351$; RO r = 0.7071, ** $p = 0.0042$).
